# Gene flow persists millions of years after speciation in *Heliconius *butterflies

**DOI:** 10.1186/1471-2148-8-98

**Published:** 2008-03-27

**Authors:** Marcus R Kronforst

**Affiliations:** 1 FAS Center for Systems Biology, Harvard University, 7 Divinity Avenue, Cambridge, MA 02138, USA

## Abstract

**Background:**

Hybridization, or the interbreeding of two species, is now recognized as an important process in the evolution of many organisms. However, the extent to which hybridization results in the transfer of genetic material across the species boundary (introgression) remains unknown in many systems, as does the length of time after initial divergence that the species boundary remains porous to such gene flow.

**Results:**

Here I use genome-wide genotypic and DNA sequence data to show that there is introgression and admixture between the *melpomene*/*cydno *and silvaniform clades of the butterfly genus *Heliconius*, groups that separated from one another as many as 30 million generations ago. Estimates of historical migration based on 523 DNA sequences from 14 genes suggest unidirectional gene flow from the *melpomene*/*cydno *clade into the silvaniform clade. Furthermore, genetic clustering based on 520 amplified fragment length polymorphisms (AFLPs) identified multiple individuals of mixed ancestry showing that introgression is on-going.

**Conclusion:**

These results demonstrate that genomes can remain porous to gene flow very long after initial divergence. This, in turn, greatly expands the evolutionary potential afforded by introgression. Phenotypic and species diversity in a wide variety of organisms, including *Heliconius*, have likely arisen from introgressive hybridization. Evidence for continuous gene flow over millions of years points to introgression as a potentially important source of genetic variation to fuel the evolution of novel forms.

## Background

Hybridization has long been recognized as an important mechanism of diversification in plants [1,2], and the exchange of genetic material via horizontal gene transfer has played a significant role in the evolution of many prokaryotic genomes [3]. In animals however, hybridization has historically been viewed as rare and evolutionarily inconsequential [4]. Despite this bias in opinion, we now know that hybridization is relatively wide-spread among animal species [5], and in some instances, it has likely had important evolutionary ramifications, such as in the origin of new species [6-10]. Surveys of hybridization in animals show that it occurs predominantly between closely-related sister species [5], and well-characterized examples of interspecific gene flow generally involve species that diverged very recently [10-13]. These observations are consistent with theory and data which show that genetic incompatibilities that result in hybrid sterility and inviability accumulate as species diverge [14]. Hybrid sterility and inviability, in turn, reduce or eliminate the opportunity for gene exchange. Despite these general trends, there are occasional examples of hybridization between distantly-related non-sister species in various animal groups [15-20]. Do these cases result in the long-term sharing of genetic material or are they simply evolutionary dead-ends?

**Figure 1:**
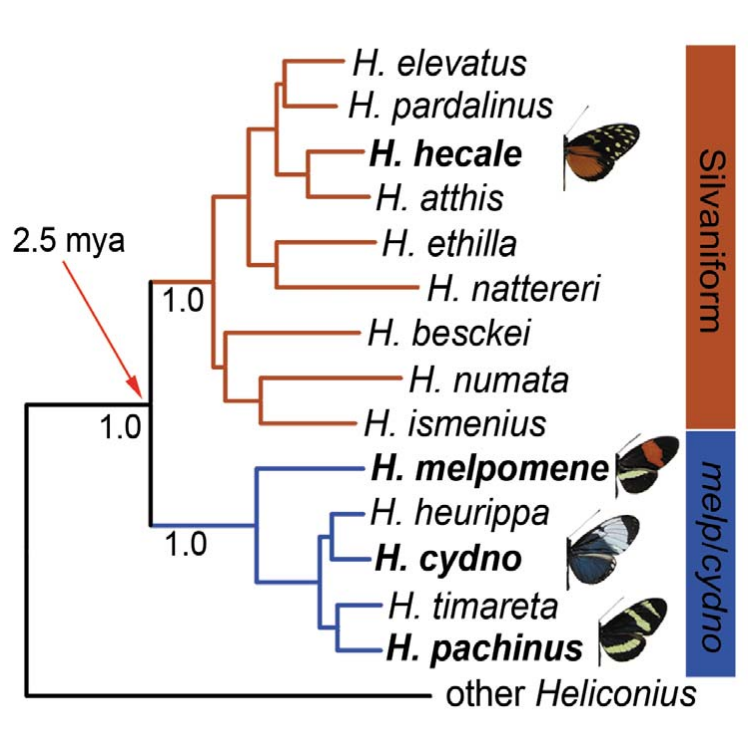
**Bayesian phylogeny showing the relationship between the distantly-related silvaniform and *melpomene*/*cydno *clades of the genus *Heliconius***. Posterior probabilities for three major nodes are shown (1.0), as are the wing pattern phenotypes for the four analyzed species. The age of the split between the *melpomene*/*cydno *and silvaniform clades was estimated assuming an evolutionary rate of 1.15% per lineage per million years [32].

To address this question I focused on the Neotropical butterfly genus *Heliconius*, a group well known for its diversity of mimetic wing patterns and for extensive hybridization [21]. As in other organisms, most hybridization in *Heliconius *occurs between closely-related species and subspecies [21]. Among these groups hybridization is known to result in gene flow [22-24]. However, members of two ecologically and morphologically distinct *Heliconius *subgroups, the *melpomene*/*cydno *clade and the silvaniform clade (Figure 1), also occasionally hybridize with one another [21,25]. In captivity, first-generation and backcross hybrids have resulted from multiple crosses between the two clades [21,26], and there are at least eleven suspected hybrids that have been collected in the field [21,25,27]. Based on phenotype and collection location, four of these field-caught specimens are believed to be hybrids between *H. melpomene *and *H. numata*, five are believed to be hybrids between *H. melpomene *and *H. ethilla*, and two are believed to be hybrids between *H. melpomene *and *H. hecale *[27]. Recent genetic data demonstrated that one of these suspected *H. melpomene*/*H. ethilla *hybrids was indeed an F_1 _hybrid [25].

While the existence of rare hybrids between the *melpomene*/*cydno *and silvaniform clades provides a potential avenue for gene flow between them, it is unknown whether introgression occurs over these large phylogenetic distances. To determine whether these distantly related groups continue to exchange genes, I used two complementary population genetic datasets to measure the extent of historical gene flow and contemporary admixture between sympatric populations of the two clades.

## Results and Discussion

### Historical migration inferred from DNA sequence data

**Figure 2:**
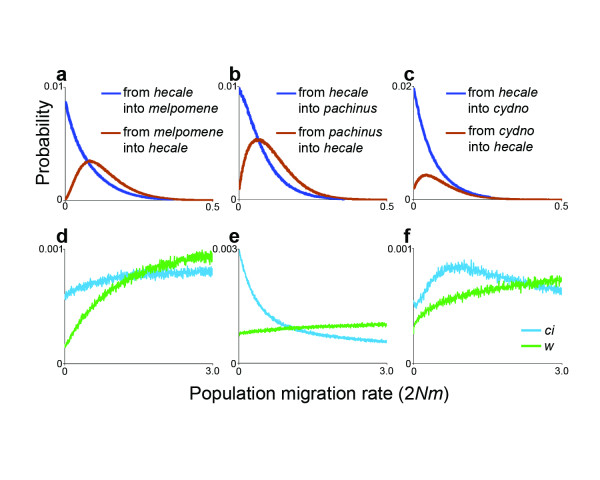
**Posterior probability distributions for between-species population migration rates**. Three pairwise comparisons based on DNA sequence data for 14 genes revealed non-zero rates of historical gene flow from the *melpomene*/*cydno *clade into the silvaniform clade (**a**, **b**, **c**). Locus specific estimates of gene flow into *H. hecale *from *H. melpomene*, *H. pachinus*, and *H. cydno*, respectively, for the two loci that exhibit evidence of between-clade introgression, *cubitus interruptus *and *white *(**d**, **e**, **f**).

In order to examine rates of gene flow between the two clades, I sequenced multiple haplotypes for one mitochondrial and 13 nuclear genes from three species in the *melpomene*/*cydno *clade (*H. cydno*, *H. pachinus*, *H. melpomene*) and one species in the silvaniform clade (*H. hecale*). I then used these data and the coalescent-based approach implemented in IM [28] to estimate the population migration rate (2*Nm*) between *H. hecale *and each of the three *melpomene*/*cydno *clade species. All three pairwise analyses revealed non-zero peaks for the marginal posterior probability distributions corresponding to the rate of historical migration from the *melpomene*/*cydno *clade into *H. hecale *(Figure 2a–c). Furthermore, for the comparisons involving *H. pachinus *and *H. melpomene*, the 90% highest posterior density interval for this function did not include zero, allowing rejection of the no gene flow hypothesis. These results, which are suggestive of unidirectional gene flow from the *melpomene*/*cydno *clade into the silvaniform clade, are consistent with the observation that of the two suspected backcross hybrids that have been collected in the field, both are believed to have resulted from backcrossing in the direction of the silvaniform clade parent (*H. melpomene *× *H. ethilla *backcrossed to *H. ethilla*) [25,27].

It is important to note that the evolutionary history of the DNA sequences studied here violates one of the underlying assumptions of the Isolation with Migration model. The IM model assumes that the two populations being examined are sister-taxa, each being more closely related to the other than either is to any other population. That is clearly not the situation with these inter-clade comparisons. However, if the sampled species provide an unbiased approximation of the genetic divergence between the two groups under study (the silvaniform and *melpomene*/*cydno *clades in this case), then it seems reasonable to model the system under the Isolation with Migration framework. The fact that analyses based on independent data (AFLPs, see below) are consistent with the IM results lends support to both the approach and the results.

### Gene flow has a variable influence across the genome

**Figure 3:**
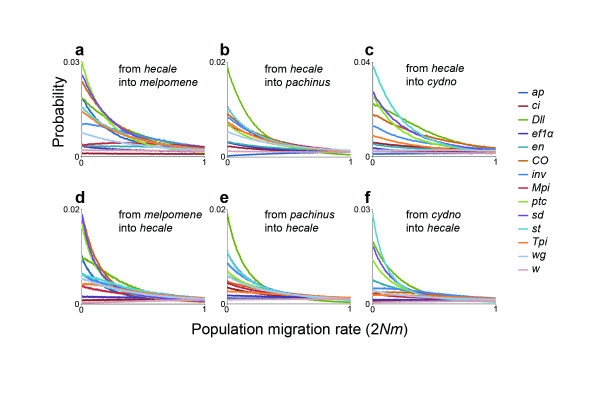
**Marginal posterior probability distributions for locus-specific migration rates**. Probability distributions for gene flow from *H. hecale *into *H. melpomene*, *H. pachinus*, and *H. cydno*, respectively (**a, b, c**). Probability distributions for gene flow into *H. hecale *from *H. melpomene*, *H. pachinus*, and *H. cydno*, respectively (**d, e, f**). Despite substantial variation in distribution shapes, most peak at or near zero suggestive of little or no gene flow.

A prediction of speciation models that permit gene flow during the process of divergence is that the influence of introgression should vary throughout the genome [29]. For instance, regions of the genome linked to loci that are under divergent selection between species should be prevented from crossing the species boundary even while other portions of the genome remain interchangeable. To test this prediction, I performed a second series of IM analyses, this time estimating separate population migration rates for each locus. Consistent with the prediction, the shapes of the migration rate posterior probability distributions varied substantially across loci. Most genes had probability distributions that peaked at or near zero indicating little or no historical gene flow (Figure 3). However, two loci, *cubitus interruptus *(*ci*) and *white *(*w*), had probability distributions consistent with gene flow from the *melpomene*/*cydno *clade into *H. hecale *(Figure 2d–f). While the migration rate probability distributions for *ci *and *w *were not well-defined, their shapes suggest that some amount of historical migration fit the data better than no migration.

**Figure 4:**
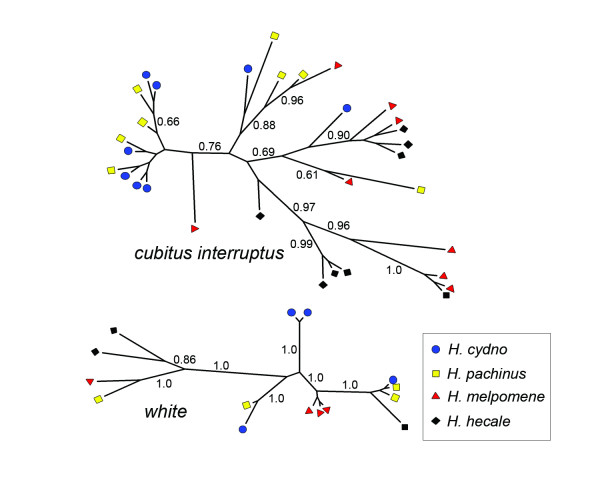
**Gene genealogies for *cubitus interruptus *and *white***. Across loci, haplotypes from *H. hecale *and the *melpomene*/*cydno *clade were reciprocally monophyletic except for at *cubitus interruptus *and *white *[24]. The lack of association between species and tree topology for these two loci is indicative of interspecific gene flow. Nodes with posterior probabilities > 0.60 are labeled.

Gene genealogies for these two loci revealed the extent of shared genetic variation between the two clades. For the other 12 loci, *H. hecale *haplotypes formed a well-supported clade that was distinct from *melpomene*/*cydno *clade haplotypes [24], but for both *ci *and *w*, *H. hecale *haplotypes were distributed across the tree (Figure 4). In addition, one identical *ci *haplotype was shared between *H. melpomene *and *H. hecale*. This haplotype was 281 bp long, 139 bp of which consisted of an otherwise highly variable intron. The sharing of an identical haplotype between these two clades is difficult to explain without recent gene flow. Previous work has found evidence of gene flow at *ci *among species within the *melpomene*/*cydno *clade [23,24] but interestingly, the locus with the clearest signature of gene flow between closely-related *Heliconius *species, *Mannose phosphate isomerase *[22,23], does not exhibit evidence of introgression over the larger phylogenetic distances examined here.

### Contemporary admixture

**Figure 5:**
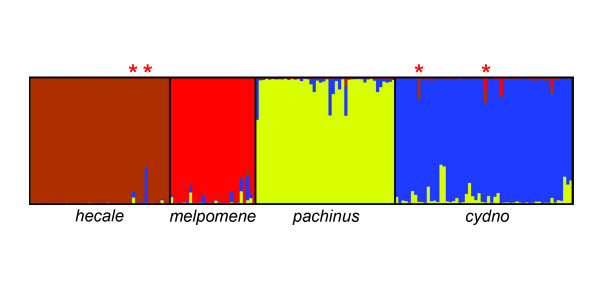
**Genetic clustering based on 520 AFLP loci**. Each individual is represented by a narrow vertical column with the proportion of the four colors indicating the genome proportion derived from each of the four populations. Two *H. hecale *and two *H. cydno *individuals exhibited evidence of mixed ancestry with the opposite clade (asterisks).

While the analyses of the DNA sequence data are consistent with a low but detectable level of gene flow between the two clades, these results do not reveal its timing. Perhaps gene flow persisted for some time after the initial divergence of the two clades but has since ceased. Alternatively, current hybridization could continue to provide a bridge between the gene pools. To test for evidence of contemporary gene flow, I genotyped 56 *H. cydno*, 44 *H. pachinus*, 27 *H. melpomene *and 44 *H. hecale *individuals at 520 polymorphic AFLP loci. Using these data and the Bayesian clustering method implemented in STRUCTURE 2.2 [30,31], I performed genetic clustering assuming four populations and allowing individuals to be of mixed ancestry. This analysis correctly delineated the four species and identified two *H. cydno *individuals and two *H. hecale *individuals with ancestry from the opposite clade (Figure 5). For each of these individuals, the 95% posterior probability interval for the genome proportion derived from the population of origin did not include one, and for three of the four individuals, the interval for the introgressed genome proportion did not include zero (Table 1). To further assess the statistical confidence for these suspected instances of admixture, I performed a second clustering analysis, this time estimating the posterior probability that each individual had pure ancestry after first indicating the population of origin for each individual and setting the prior probability of pure ancestry to 0.95. All four of the individuals identified in the first analysis had low probabilities of pure ancestry and high probabilities of mixed ancestry with the other clade (Table 1), supporting the hypothesis of contemporary admixture. Interestingly, the four individuals with recently-mixed ancestry based on the AFLP data were not included in the DNA sequence analyses so the signatures of gene flow in the two datasets are independent of one another.

**Table 1:** AFLP based estimates of mixed ancestry. Admixture proportions (with 95% confidence intervals) and ancestry probabilities for the four individuals that exhibited evidence of between-clade mixed ancestry.

Individual	Proportion of genome derived from:				Probability of pure ancestry	Probability of mixed ancestry with the opposite clade
			
	*H. hecale*	*H. melpomene*	*H. pachinus*	*H. cydno*		
*hecale *#33	0.898 (0.791–0.992)	0.005 (0.000–0.064)	0.045 (0.000–0.159)	0.052 (0.000–0.199)	0.211	0.789
*hecale *#37	0.709 (0.594–0.818)	0.006 (0.000–0.072)	0.002 (0.000–0.024)	0.283 (0.167–0.400)	0.000	1.000
*cydno *#8	0.172 (0.092–0.262)	0.001 (0.000–0.015)	0.011 (0.000–0.143)	0.816 (0.674–0.905)	0.000	1.000
*cydno *#29	0.096 (0.009–0.186)	0.098 (0.000–0.224)	0.005 (0.000–0.060)	0.801 (0.653–0.937)	0.169	0.626

### Gene flow has persisted for millions of years after speciation

Together, these population genetic data are consistent with a history of divergence with gene flow between the *melpomene*/*cydno *and silvaniform clades. Average pairwise mtDNA divergence between these two groups is 5.7% (SE = 0.5%). Using the estimate of 1.1 – 1.2% divergence per lineage per million years [32], this equates to approximately 2.5 million years of divergence. With a minimal *Heliconius *generation time of one month, this represents as many as 30 million generations of evolution along each lineage since the speciation event that precipitated cladogenesis.

## Conclusion

These data suggest that the process of divergence that ultimately results in reproductively isolated species can be prolonged. The fact that genomes can remain open to gene flow very long after the speciation process is initiated greatly expands the evolutionary novelty that can be generated from introgression. Some portion of the phenotypic and species diversity in *Heliconius *has very likely arisen from introgressive hybridization [6,26]. The results presented here suggest that the *melpomene*/*cydno *and silvaniform clades of *Heliconius *have experienced continuous gene flow over millions of years. Thus, introgression has had the potential to provide a ready source of genetic variation to fuel this expansive adaptive radiation. For many organisms, even rare hybridization with distantly related species may allow for the continued exchange of genetic material which may serve as a long-term source of variation for adaptive change.

## Methods

### Samples

I collected 56 *H. cydno*, 44 *H. pachinus*, 27 *H. melpomene*, and 44 *H. hecale *individuals from various locations throughout Costa Rica. None of the sampled individuals exhibited phenotypic evidence of introgression from the opposite clade. All specimens were collected as adults in the summer of 2000 and 2002. Tissue was preserved in 95% ethanol and DNA was extracted with a DNeasy Tissue Kit (Qiagen) or using a phenol/chloroform extraction protocol.

### DNA sequencing and analysis

I sequenced multiple haplotypes for one mitochondrial locus and 13 nuclear loci from the four species using primers and methods described previously [24,33]. The loci were *apterous *(*ap*), *cubitus interruptus *(*ci*), *cytochrome oxidase I *&*II *(*CO*, mtDNA), *Distal-less *(*Dll*), *elongation factor 1α *(*ef1α*), *engrailed *(*en*), *invected *(*unv*), *Mannose phosphate isomerase *(*Mpi*), *patched *(*ptc*), *scalloped *(*sd*), *scarlet *(*st*), *Triose phosphate isomerase *(*Tpi*), *white *(*w*), and *wingless *(*wg*). I also sequenced portions of the genes *cinnabar *[24] and *decapentaplegic *[34] but these loci were excluded from the analyses because only one *H. hecale *sequence was obtained for each. All sequences have been deposited in GenBank under accession numbers AY744577–AY744672, AY745254–AY745278, AY745315–AY745335, AY745356–AY745490, DQ448305–DQ448516 and EF041105–EF041122. For analysis, the datasets for *CO*,*ef1α*,*Mpi*, and *Tpi *were supplemented with published sequences from GenBank [24].

I used the comparative DNA sequence data and the Isolation with Migration model implemented in IM [28] to infer historical rates of between-species gene flow. IM cannot handle alignment gaps or evidence of recombination in DNA sequence data. Therefore, I removed gaps and searched for evidence of recombination using the four-gamete test in DnaSP 3.5 [35]. For those loci that exhibited evidence of recombination, the sequence alignment was divided into portions that showed no evidence of recombination and only one portion was included in the analysis. In an effort to preserve as much genealogical information as possible, the portion with the most polymorphisms was chosen. The size of these non-recombining portions ranged from 60 bp for *st *in the *H. cydno*/*H. hecale *comparison to 1111 bp for *wg *in the *H. melpomene*/*H. hecale *comparison.

For each comparison between *H. hecale *and one of the three *melpomene*/*cydno *clade species, I ran IM in two different ways. First, I used the data for all loci to estimate a single pair of bidirectional population migration rates (Figure 2a–c). Then, using the same dataset, I allowed each locus to have a separate pair of population migration rates (Figure 3). For each analysis, IM was run with 10 Metropolis-coupled chains for 300,000 burn-in steps followed by 2 × 10^7^steps of data collection. Following other implementations of IM [28], I used the HKY model for the mitochondrial locus and the infinite-sites model for the nuclear loci.

### Phylogeny estimation

I used published DNA sequences from six genes [36] and MrBayes [37] to estimate a phylogeny of the silvaniform and *melpomene*/*cydno *clades of *Heliconius*. Four Metropolis-coupled Markov chains were run for 250,000 burn-in generations followed by 1.75 × 10^6 ^generations of data collection. The age of the *melpomene*/*cydno *and silvaniform split was estimated based on 1606 base pairs of mtDNA from ref. 36, assuming an evolutionary rate of 1.15% per lineage per million years [32]. Gene genealogies for *ci *and *w *were also estimated using MrBayes, based on the GTR+I+Γ model of molecular evolution.

### AFLP genotyping and analysis

Using the ABI Plant Mapping Kit (PE Applied Biosystems), I genotyped each individual with four selective AFLP primer combinations; EcoRI-ACT/MseI-CAT, EcoRI-ACT/MseI-CTG, EcoRI-ACA/MseI-CAT, and EcoRI-ACA/MseI-CTG. AFLP fragments were separated using an ABI 3100 automated genotyper and then scored using ABI GENEMAPPER software version 3.7. In total, each individual was typed for the presence or absence of a fragment at 925 AFLP loci. In an effort to focus on the most informative and reliable markers, only the 520 AFLPs that had a minor allele frequency ≥ 0.05 were used in further analyses.

I used the AFLP data and the Bayesian-based genetic clustering program STRUCTURE 2.2 [30,31] to define populations and estimate admixture among them. The data were analyzed in two ways. First, I performed naïve admixture clustering assuming four populations. As part of this analysis, I estimated the 95% posterior probability interval around each individual admixture proportion. Second, I performed an additional round of admixture clustering after first indicating the population of origin for each individual and setting the prior probability of pure ancestry to 0.95. As part of this analysis, I estimated the posterior probability that each individual was misclassified or had an ancestor from each of the other species within the last three generations. For both analyses, STRUCTURE was run for 10,000 burn-in generations followed by 10^6 ^generations of data collection.

## Authors' contributions

MRK conceived of the study, performed the research, analyzed the data, and wrote the manuscript.
